# Exploring the measurement of psychological resilience in Chinese civil aviation pilots based on generalizability theory and item response theory

**DOI:** 10.1038/s41598-024-52229-7

**Published:** 2024-01-22

**Authors:** Yanzeng Zhao, Keyong Zhu, Jun Zhang, Ziyu Liu, Lijing Wang

**Affiliations:** 1 Fundamental Science on Ergonomics and Environment Control Laboratory, School of Aeronautic Science and Engineering, Beihang University, Beijing, 100191 China; 2 Beijing Advanced Innovation Centre for Biomedical Engineering, School of Engineering Medicine, Beihang University, Beijing, 100191 China

**Keywords:** Human behaviour, Psychology, Risk factors, Aerospace engineering

## Abstract

Understanding and accurately measuring resilience among Chinese civil aviation pilots is imperative, especially concerning the psychological impact of distressing events on their well-being and aviation safety. Despite the necessity, a validated and tailored measurement tool specific to this demographic is absent. Addressing this gap, this study built on the widely used CD-RISC-25 to analyze and modify its applicability to Chinese civil aviation pilots. Utilizing CD-RISC-25 survey data from 231 Chinese pilots, correlational and differential analyses identified items 3 and 20 as incongruent with this population's resilience profile. Subsequently, factor analysis derived a distinct two-factor resilience psychological framework labeled “Decisiveness” and “Adaptability”, which diverged from the structure found in American female pilots and the broader Chinese populace. Additionally, to further accurately identify the measurement characteristics of this 2-factor measurement model, this study introduced Generalized Theory and Item Response Theory, two modern measurement analysis theories, to comprehensively analyze the overall reliability of the measurement and issues with individual items. Results showed that the 2-factor model exhibited high reliability, with generalizability coefficient reaching 0.89503 and dependability coefficient reaching 0.88496, indicating the 2-factor measurement questionnaire can be effectively utilized for relative and absolute comparison of Chinese civil aviation pilot resilience. However, items in Factor 2 provided less information and have larger room for optimization than those in Factor 1, implying item option redesign may be beneficial. Consequently, this study culminates in the creation of a more accurate and reliable two-factor psychological resilience measurement tool tailored for Chinese civil aviation pilots, while exploring directions for optimization. By facilitating early identification of individuals with lower resilience and enabling the evaluation of intervention efficacy, this tool aims to positively impact pilot psychological health and aviation safety in the context of grief and trauma following distressing events.

## Introduction

Resilience, recognized as an essential trait enabling individuals to navigate the adversities of life, plays a pivotal role in the context of grief and trauma following distressing events^1,2^. Defined as a stable, adaptive, and inherent characteristic, resilience equips individuals with specific skills, such as active problem-solving, essential for maintaining psychological and emotional equilibrium amidst stressful or traumatic experiences^2,3^. Research consistently indicated a strong correlation between resilience levels and various outcomes, encompassing emotional well-being, coping strategies, and the impact on mental health following encounters with distressing events^4,5^. In the face of grief and trauma resulting from events such as loss, accidents, or natural disasters, an accurate tool for measuring resilience becomes increasingly significant. This precise measurement instrument holds immense value in identifying individuals experiencing insufficient resilience following distressing events, allowing for timely and targeted interventions to mitigate the impact of trauma and grief. Moreover, it serves as a quantitative means to evaluate the efficacy of interventions, verifying the restoration of resilience to a level conducive to effective stress management in the aftermath of trauma. As the impact of distressing events on mental health and well-being becomes more evident, the need for a reliable resilience assessment tool has garnered significant attention, particularly in industries where individuals are routinely exposed to traumatic incidents.

The Connor–Davidson Resilience Scale (CD-RISC-25), developed by Connor and Davidson^6^, is currently one of the most widely utilized measures of resilience^7^, which was designed to assess a series of trait characteristics that are understood to constitute trait resilience^8^. The CD-RISC-25 has been believed to be related to several psychological and behavioral domains and extensively implemented among various groups including community residents, staff, university students, and psychiatric outpatients^9–15^. Originally designed based on samples from the American general population, primary care, and four other groups in the United States, the CD-RISC consists of 25 items and is divided into five dimensions: personal competence, high standards, and tenacity (8 items); trust or tolerance of negative affect and stress (7 items); acceptance of change and secure relationships (5 items); control (3 items); and spirituality (2 items)^6^.

As with all questionnaire measures, the quality of the CD-RISC-25 may be influenced by cultural variations and differences in the characteristics of the surveyed populations when applied across different countries or demographic groups. If the questionnaire items do not accurately, consistently, and stably capture the experiences of respondents, the credibility of the results will be compromised, negatively impacting subsequent data analysis and conclusion generalization^9,16–21^. Abundant research consistently showed that resilience is a complex construct involving various aspects of adjustment and adaptation. However, several studies have revealed a lack of strong replication regarding the original factor structure of the CD-RISC-25^8,17,22^. Specifically, the retained items and the delineation of psychological structures exhibit notable variation across populations within the same country. For instance, in the case of Chinese military personnel^23^, items 3, 15, 18, and 20 were omitted, leading to the emergence of a three-factor structure encompassing Competency, Toughness, and Adaptability. In contrast, among Chinese employees^24^, all items were retained, yielding a four-factor structure that encapsulates Tolerance for Stress, Tenacity, Adaptability, and Optimism. Furthermore, the reliability analyses conducted within the same population can yield differing results across cultural contexts. While Connor and Davidson identified a five-factor structure in American adults^6^, Yu et al. discerned a three-factor structure comprising Resilience, Strength, and Optimism in China^17^. Evidently, the application of the CD-RISC-25 across various countries and populations necessitates thorough reliability analyses to discern pertinent items, delineate psychological constructs accurately, and ensure representation that aligns with the target sample, thereby enhancing measurement precision.

Pilots in their professional careers may encounter various traumatic events or adversities, some of which may pose potential threats to flight safety^25^. Firstly, extreme weather conditions, sudden meteorological changes, or mechanical failures may trigger emergencies during flight, necessitating rapid decision-making by pilots to ensure the safety of the crew and passengers. Additionally, unconventional flight conditions such as emergency landings, stalls, and system malfunctions can present significant challenges to pilots' psychological and technical capabilities in the rapidly changing aviation environment. On a more personal level, pilots may face life upheavals such as family issues, job insecurity, or major life events, all of which can have negative impacts on their psychological state and focus. Mental health issues, such as work-related stress, anxiety, and depression, may also interfere with pilots' decision-making abilities and coping mechanisms, thereby posing a potential threat to flight safety. In the face of these adversities and traumatic events, pilots need to demonstrate a high degree of resilience and adaptability to effectively manage and maintain the safety of flight operations. Currently, the CD-RISC-25 has been utilized to assess resilience levels in female civilian pilots in the United States and military aviation pilots in China. In reliability analyses of the CD-RISC-25 for American female civilian pilots^25^, items 2, 3, 6, 7, and 9 were omitted, revealing a two-factor model comprised of "competence and adaptability" and "adaptability and perseverance". In contrast, when analyzing Chinese military pilots^26^, only item 3 was removed, resulting in identification of a five-factor model. Clearly, despite both assessing CD-RISC-25 reliability in pilots, the American sample deleted 5 items and obtained a two-factor structure, while the Chinese sample deleted just one item and obtained a five-factor structure. Significant differences existed between the two countries regarding the number of omitted items and resultant factor structures. Given the critical importance of accurately measuring civil aviation pilots' psychological resilience for aviation safety, and accounting for disparities in work environments, duties, and pressures between Chinese and American pilots and military and civilian pilots, it is vital to collect CD-RISC-25 data from Chinese civil aviation pilots and conduct corresponding reliability analyses, which will enhance precision in assessing resilience levels among this population of Chinese civil aviation pilots.

Moreover, the examination of CD-RISC-25 reliability has predominantly relied on traditional Classical Test Theory (CTT) methodologies^8,25^. While CTT provides conventional reliability assessments, it falls short in accounting for relevant sources of measurement error^27^. Consequently, CTT offers an incomplete representation of score dependability^28,29^, potentially leading to issues such as inaccurate or unstable measurement outcomes^28,30,31^. Therefore, to effectively assess the psychological resilience levels of Chinese pilots, improved reliability analysis tools are essential. Generalizability Theory (GT), proposed and implemented by Cronbach et al. in the 1970s^32^, was established to address the limitations of CTT. Taking a comprehensive view, GT employs mathematical modeling and statistical calibration to thoroughly dissect diverse sources of variance. Specifically, GT breaks down the total variance into distinct components representing the target construct versus error^33^. Subsequently, GT estimates the relative magnitudes of these variance components using analysis of variance (ANOVA) techniques. Thus, GT can elucidate the contributions of specific facets or errors to measurement inaccuracy, mitigating inaccuracies stemming from imperfect CTT reliability assessments^33,34^. Due to its ability to account for diverse sources of error, GT has been extensively applied in various assessments^35–38^. Furthermore, Multivariate GT extends the capabilities of GT when dealing with multidimensional instruments by considering covariance and applying identity roots. This is especially pertinent when assessing multidimensional constructs like resilience. Additionally, GT not only allows for evaluating the effectiveness of measurement results for norm-referenced or criterion-referenced purposes, complementing CTT in this regard, but it also permits the investigation of reliability fluctuations under altered test conditions. This includes scenarios such as modifications to questionnaire length. Such flexibility not only facilitates optimal error control but also provides valuable insights to guide design decisions and enhance the precision of measurement^30,39^.

Furthermore, as the relationship between resilience and external behaviors is predominantly nonlinear, the utilization of Item Response Theory(IRT) analysis becomes essential^40–42^. This analytical approach elucidates how item response outcomes are influenced by the combined effects of individual ability levels and item characteristics. By leveraging the participants' actual responses to each item, the IRT employs the item characteristic function—reflecting the likelihood of participant responses based on varying abilities for a particular item or option—as its framework^43^. Through this method, it estimates the levels of potential mental traits and the metrological parameters of items. Consequently, this approach not only aids in estimating subjects' resilience but also discerns the specific quality of each item, laying a foundation for item deletion in psychometric questionnaires^44,45^. While the IRT analysis in CD-RISC-25 demonstrates substantial utility, it has only been applied once within the Spanish general population^40^. To more effectively assess the resilience of civil aviation pilots and develop a tailored measurement tool for this demographic, this study endeavors to introduce an in-depth IRT analysis to scrutinize the measurement quality of each question in detail.

In conclusion, this research utilized advanced measurement analysis theories, specifically Multivariate Generalizability Theory and Item Response Theory, to thoroughly examine the CD-RISC-25 scale among Chinese civil aviation pilots, considering their exposure to disaster events. Apart from identifying inconsistent items, the study aimed to reveal a tailored resilience model that suits the psychological profile of these pilots. Through these methodologies, the investigation delved into the subtleties of resilience measurement, offering insights into reliability, structural elements, and potential improvements within the adapted tool. By developing a customized and robust measurement model for assessing the resilience of Chinese civil aviation pilots, this study not only enhances theoretical understanding but also presents methodological advancements in evaluating and fortifying resilience, especially post-distressing events. This, in turn, shows promise for refining practices and interventions in grief and trauma, notably in professions where individuals frequently face potentially traumatic incidents.

## Methods

### Participants

An online questionnaire was administered to pilots participating in recurrent training. Ethical approval for this research was obtained from the Ethics Committee of Aeronautical Science and Engineering (ASE) and all methods were performed in accordance with the relevant guidelines and regulations. Prior to the administration of the test, flight instructors explained the purpose of the questionnaire assessment to the participants, provided standardized instructions, and informed them of the confidentiality principles. After obtaining informed consent from the pilots, the questionnaires were completed. Data collection for the questionnaires took place from September 16, 2023, to October 29, 2023, resulting in a total of 316 responses. Following the selection rule of "Please select 4 for this item" and the exclusion of responses where all items had consistent scores, invalid questionnaires were removed. As a result, a total of 231 valid questionnaires were ultimately confirmed. All participants were male due to the very low proportion of female pilots in China^46^.

### Materials

#### Connor Davidson resilience scale

The Connor–Davidson Resilience Scale (CD-RISC), developed by Connor and Davidson, is a psychological assessment tool consisting of 25 items^6^. In the current measurement, a Likert 5-point scale was used for evaluation, ranging from 0, indicating "completely disagree", to 4, signifying "strongly agree". Scores on this scale range from 0 to 100, with higher scores indicating higher levels of psychological resilience. For this measurement, the Chinese translation version introduced by YU Xiaonan was selected^17^.

### Statistical analyses

The analysis of item correlation, discriminant analysis, factor analysis, and demographic analysis of participants were conducted using SPSS22 and SPSSAU 23.0 (Statistical Product and Service Software Automatically), which is a data science analysis platform that has been extensively utilized across various domains in natural sciences, technological sciences, and social sciences, including but not limited to education, pedagogy, psychology, medicine, management, and economics and finance^47^. The multivariate generalizability analysis was completed using the mGENOVA software developed by Brennan^48^, and Item Response Theory Analysis was conducted using the mirt package^49^ in R (version 4.3.1).

#### Item correlation analysis

According to the requirements of psychometrics, it is ideal for each item to have a pearson correlation coefficient with the total score of not less than 0.3^50^. When the correlation coefficient exceeds 0.4, the item's performance is considered quite satisfactory.

#### Item discriminant analysis

Based on the total scores obtained from the 25-item CD-RISC scale, participants were selected for discriminant analysis. The top 27% and bottom 27% of the total score distribution were categorized as the high-scoring group and low-scoring group, respectively^50^. To assess discriminant validity, a t-test was conducted to determine whether the differences observed between these groups were statistically significant.

#### Factor analysis

For factor analysis, this study initially conducted confirmatory factor analysis (CFA) on the two-factor structure among female American pilots and the three-factor structure within the general Chinese population, aiming to explore the model's goodness of fit. In cases where the model's fit is inadequate, oblique rotation will be employed as previous research had shown that resilience factors have moderate-to high correlations for Exploratory Factor Analysis (EFA) to unveil the resilience structure among Chinese civil aviation pilots. Model acceptance or rejection was determined based on the following fit indices: Comparative Fit Index (CFI), Adjusted Goodness of Fit Index (GFI), Tucker–Lewis Index (TLI), and Root Mean Square Error of Approximation (RMSEA). Established criteria for satisfactory model fit included CFI ≥ 0.90, GFI ≥ 0.90, TLI ≥ 0.90, and RMSEA ≤ 0.1^51^.

#### Multivariate generalizability analysis

Based on the results of the factor analysis described above, a single-facet crossed design (p × i multivariate generalizability model) was implemented, where p represented the pilots, and i represented the items. Utilizing the multivariate generalizability model, data were collected and analyzed for G-study and D-study to assess the reliability and generalizability of the results. In G-study, three variance components were estimated as part of this design: pilots (p), items (i), and pilots by items (pi). In evaluative settings, large pilots score variance is almost always desired as the variability in scoring can be attributed to the test-takers’ differing levels of proficiency for a certain construct. At the same time, less measurement error due to facets as items is usually desirable. G-studies are followed by decision (D) studies which utilize variance estimates obtained from G-study procedures to estimate generalizability (G coefficients or ερ^2^) and dependability (phi coefficients or Φ) coefficients. The G coefficient can be used to evaluate the reliability of the norm-referenced test and to compare the resilience of pilots. The phi coefficients are used to assess the reliability of the standard reference test and can be used to assess the absolute value of pilots' resilience.

#### Item response theory analysis

Based on the outcomes of factor analysis, the study employed the 2-parameter graded response model (GRM) to scrutinize each item's properties concerning the evaluation of Chinese civil aviation pilots^52^. This investigation focused on assessing the item discrimination parameter (a) and the location parameter (bi) for each item. The discrimination parameter (a) is pivotal in gauging an item's efficacy in distinguishing between various levels of the latent trait, serving as a fundamental measure of its capacity to differentiate among individuals with diverse degrees of resilience. Besides, the location parameter (bi) delineates the difficulty of an item in generating a 50% probability of a correct response at a specific ability level. Additionally, the research calculated both the test information of each item and the cumulative information of the entire measurement. Test information functions (TIFs) were utilized to illustrate the information and standard error conferred by the test or an item across different ability levels, aiding in identifying the optimal range of the latent trait measured by an item or a test. As per Baker and Kim's categorization, discrimination parameter (a) values were classified into five levels: very low (0.01–0.34), low (0.35–0.64), moderate (0.65–1.34), high (1.35–1.69), and very high (1.70 or higher)^45,53^. Concerning the location parameter (bi), it was crucial to exhibit a systematic ordering of the difficulty parameters to aid in assessing abilities. The determination to eliminate an item relied on an evaluation of the discrimination parameter (a), the location parameter (bi), and the test information of the item.

#### Demographics analysis

Utilizing the outcomes of factor analysis, IRT was employed to derive individual resilience scores for each pilot across different factors. Due to the non-normal distribution of the data, median CD-RISC scores were computed for each group, and the Wilcoxon Rank Sum test was utilized to perform pairwise comparisons. This methodology enables a thorough exploration of potential demographic variations, fostering a comprehensive insight into the psychological resilience of pilots as addressed within this study.

## Results

### Descriptive statistics

The lowest option for items 10, 13, and 19 was 1, indicating "rarely true". Except for items 10, 13, and 19, all options for each item had been chosen. In the average scores calculated from 25 items, item 10 stood out with the highest average score of 3.329, signifying a frequency from "often true" to "true nearly all the time". Following closely behind were items 9 and 2. Conversely, items 20 and 3 received the lowest average scores, both falling below 2, which suggested they were considered "rarely true".

### Results of item correlation analysis

Pilots' overall scores were tallied from their responses to the 25 items, and the Pearson correlation coefficients between each item's score and the total score were calculated. With the exception of items 3 (coef. = − 0.049) and 20 (coef. = 0.065), which display an insignificant correlation with the total score, all other items demonstrate correlations surpassing 0.4 (p < 0.01). As a result, it was advisable to exclude items 3 and 20 from the questionnaire in the resilience assessment of Chinese civil aviation pilots.

### Results of item discriminant analysis

Following the 27% selection criterion, there were a total of 62 pilots in the low-scoring group and 62 pilots in the high-scoring group. A t-test was conducted for the remaining 23 items, indicating that all 23 remaining items should be retained for further analysis (p < 0.01).

### Results of factor analysis

To explore whether the measurement model constructed by other populations is applicable to Chinese male pilots, initially, a goodness-of-fit analysis was carried out for the two-factor model exhibited by resilience in female pilots in the United States^25^ and the three-factor model demonstrated by the general population in China^17^ based on the collected data in this study. As shown in Table 1, it was indicated by the results that the requirements of model fit were not fully met by either. However, a relatively better model fit was shown by the two-factor model of resilience in American female pilots. Consequently, an exploratory factor analysis would be proceeded with.

**Table 1:** Model fit analysis of resilience structure.

	GFI	CFI	TLI	RMSEA
Criteria	> 0.9	> 0.9	> 0.9	< 0.1
Resilience factor structural model of U.S. female pilots	0.848	0.908	0.897	0.069
Resilience factor structural model of Chinese people	0.800	0.861	0.847	0.075

**Figure 1:**
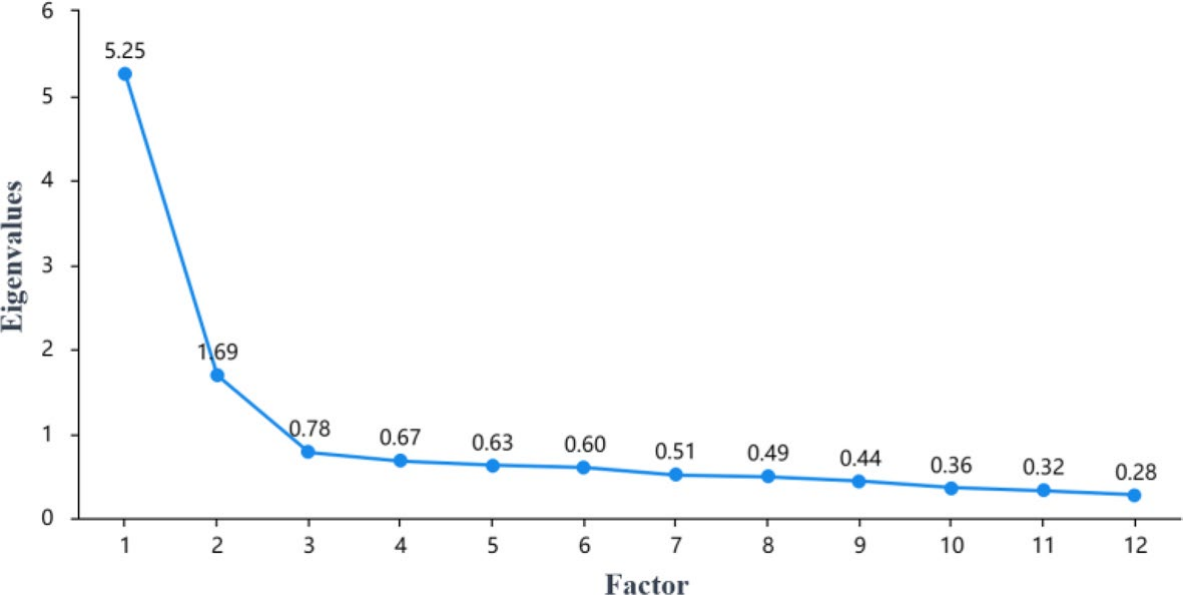
Scree plot.

Before conducting the exploratory factor analysis (EFA), Bartlett's sphericity test was used to confirm the feasibility of the analysis. The Kaiser–Meyer–Olkin (KMO) measure, which surpassed the critical criterion of 0.8, was observed at 0.942. Additionally, Bartlett's sphericity test yielded a χ^2^ value of 2641.776 (df = 253, p = 0.000). Collectively, these results suggested that the data collected for this study fulfilled the necessary conditions for carrying out an exploratory factor analysis. The result of the screen plot is shown in Fig. 1, which shows that two factors can be extracted. Following the guideline of utilizing factor loading coefficients greater than 0.4 and communalities greater than 0.4 in the EFA, a total of 11 items—specifically, 4, 6, 8, 11, 12, 13, 14, 16, 21, 22, and 24—were eventually eliminated after two rounds of factor analysis with the factor load shown in Table 2. Their respective communalities were below 0.4, indicating a notably weak relationship between the factors and the research items. Consequently, the factors were unable to effectively extract information from the research items. Subsequent to their removal, another round of EFA was conducted, resulting in a two-factor model. The variance explained by these two rotated factors was 35.364% and 22.245%, respectively, with a cumulative variance explained after rotation at 57.609%.

**Table 2:** Factor load of resilience structure for Chinese pilots.

	Factor Loading		Communality
	Factor 1	Factor 2	
5	0.488	0.457	0.447
7	0.646	0.212	0.462
15	0.679	0.051	0.463
17	0.834	− 0.059	0.699
18	0.799	− 0.07	0.644
19	0.708	0.001	0.501
23	0.847	− 0.252	0.78
25	0.722	0.072	0.527
1	0.153	0.721	0.543
2	− 0.032	0.688	0.474
9	− 0.152	0.851	0.748
10	− 0.082	0.786	0.624

In the resulting two-factor model from the factor analysis, Factor 1 encompassed the following items: 5, 7, 15, 17, 18, 19, 23, and 25, while Factor 2 included items: 1, 2, 9, and 10.

### Results of multivariate generalizability analysis

#### G-study

Based on the research design involving 2 latent factors derived from factor analysis, the mGENOVA software was employed to obtain estimates of the variance and covariance components for the interaction effects between pilots (p), questionnaire items (i), and the pilot-questionnaire item interactions (pi) across the two factors. The data presented in Table 3 highlights the substantial variance arising from pilots and their interactions with items across both factors, indicating a significant origin of variability. In contrast, the variance stemming solely from the items was minimal, implying a restricted level of measurement error linked directly to the items. Additionally, the correlation and covariance coefficients revealed a high level of consistency among the factors, showing that pilots' scoring patterns across various factors closely match their scoring trends in other factors.

**Table 3:** Estimated G study variance and covariance components.

Effect	Factor1	Factor2
p	0.27949	0.56854
	0.12705	0.17867
i	0.03981	–
	–	0.01217
pi	0.30442	–
	–	0.23783

Lower diagonal elements are covariances. Upper diagonal elements are correlations.

#### D-study

Derived from the variance and covariance matrix estimated through the G-study, the variance components for pilots' overall scores across the two factors, along with the respective error variance components, were further estimated. This enabled the computation of generalizability coefficients and phi coefficient. Table 4 reveals that the variance component for Factor 1 in the overall score is larger (0.27949) than that of Factor 2 (0.17867). Considering the error components, Factor 1 exhibited higher reliability, leading to a larger generalizability coefficient (Gen = 0.88017), which implied its suitability for norm-referenced assessments used for relative decision-making. For criterion-referenced assessments, Factor 1 (phi = 0.86659) still maintained superior measurement precision, demonstrating comparable levels of reliability.

**Table 4:** D study results for individual variables.

	Factor1	Factor2
Univ score var	0.27949	0.17867
Gen coefficient (ερ^2^)	0.88017	0.75031
Phi coefficient (Φ)	0.86659	0.74084

By allocating weights in accordance with the proportion of test items within each section, the Generalizability Coefficients for the two factors were amalgamated, yielding the Universe score's variance and estimates for corresponding error variance components. Subsequently, the Generalizability Coefficient for the comprehensive domain score could be ascertained. It is evident in Table 5 that the Composite Generalizability Coefficient stood at 0.89503, while the Composite Phi was 0.88496, both indicating highly favorable outcomes. Moreover, the Composite Relative Error Variance registered at 0.02352, and the Composite Absolute Error Variance was a mere 0.02607, both of which were notably low.

**Table 5:** Composite generalizability and composite Phi coefficients in D study.

	Results
Composite universe score variance	0.20054
Composite relative error variance	0.02352
Composite absolute error variance	0.02607
Composite error variance for mean	0.00352
Composite generalizability coefficient (ερ^2^)	0.89503
Composite Phi coefficient (Φ)	0.88496

Seen in Table 6, the items within factor 1 demonstrated higher reliability compared to those in factor 2 when weighted by the number of items. This indicates that, overall, factor 1 performed better than factor 2 in this resilience measure. Factor 2 necessitated the inclusion of a greater number of items to attain a similar measurement effect as factor 1. In simpler terms, items in factor 2 might pose more challenges in measurement.

**Table 6:** Factor weights in composite scores and proportional contribution to reliability.

	Factor 1	Factor 2
w-weights	0.6667	0.3333
Comp univ score var	76.02%	23.98%

When adjusting the quantity of items within Factor 1, it is observed from Table 7 that, when Factor 1 maintains more than 5 items, its generalizability coefficient and phi coefficient remain above 0.8, demonstrating good measurement performance.

**Table 7:** Changes in reliability coefficients with varying quantity of Factor 1 items.

Quantity of items in Factor 1	Gen coefficient of Factor 1	Phi coefficient of Factor 1	Comp Gen coefficient	Comp Phi coefficient
4	0.78598	0.76458	0.84010	0.82742
5	0.82113	0.80236	0.85786	0.84582
6	0.84636	0.82969	0.87256	0.86119
7	0.86535	0.85038	0.88478	0.87408
8	0.88017	0.86659	0.89503	0.88496

### Results of item response theory analysis

The item parameters were summarized in Table 8, which were obtained by using the mirt package. The discrimination parameters (a) for all items ranged from 1.718 to 2.921, meeting the standard of greater than 1.7. This indicated that all items exhibit strong discrimination, with the items within Factor 1 demonstrating relatively higher discrimination.

**Table 8:** Item parameter estimates in the graded response model.

	Item	a	b1	b2	b3	b4	Item information
Factor1	5	2.276	− 3.147	− 2.56	− 1.242	1.12	6.89
	7	2.199	− 2.81	− 1.525	− 0.312	1.819	7.31
	15	1.944	− 3.371	− 1.926	− 0.384	1.676	6.16
	17	2.921	− 3.31	− 2.116	− 0.839	1.204	10.38
	18	2.237	− 3.226	− 2.021	− 0.56	1.451	7.39
	19	2.067	–	− 2.36	− 1.12	1.56	5.43
	23	1.775	− 2.547	− 1.483	− 0.091	2.118	5.37
	25	2.269	− 2.616	− 2.113	− 0.854	1.217	6.82
Factor2	1	2.707	− 3.443	− 2.765	− 1.513	1.137	8.62
	2	1.718	− 4.014	− 2.85	− 1.698	0.685	4.38
	9	1.948	− 3.401	− 2.9	− 1.827	0.533	5.09
	10	1.913	–	− 3.401	− 2.037	0.329	4.58

**Figure 2:**
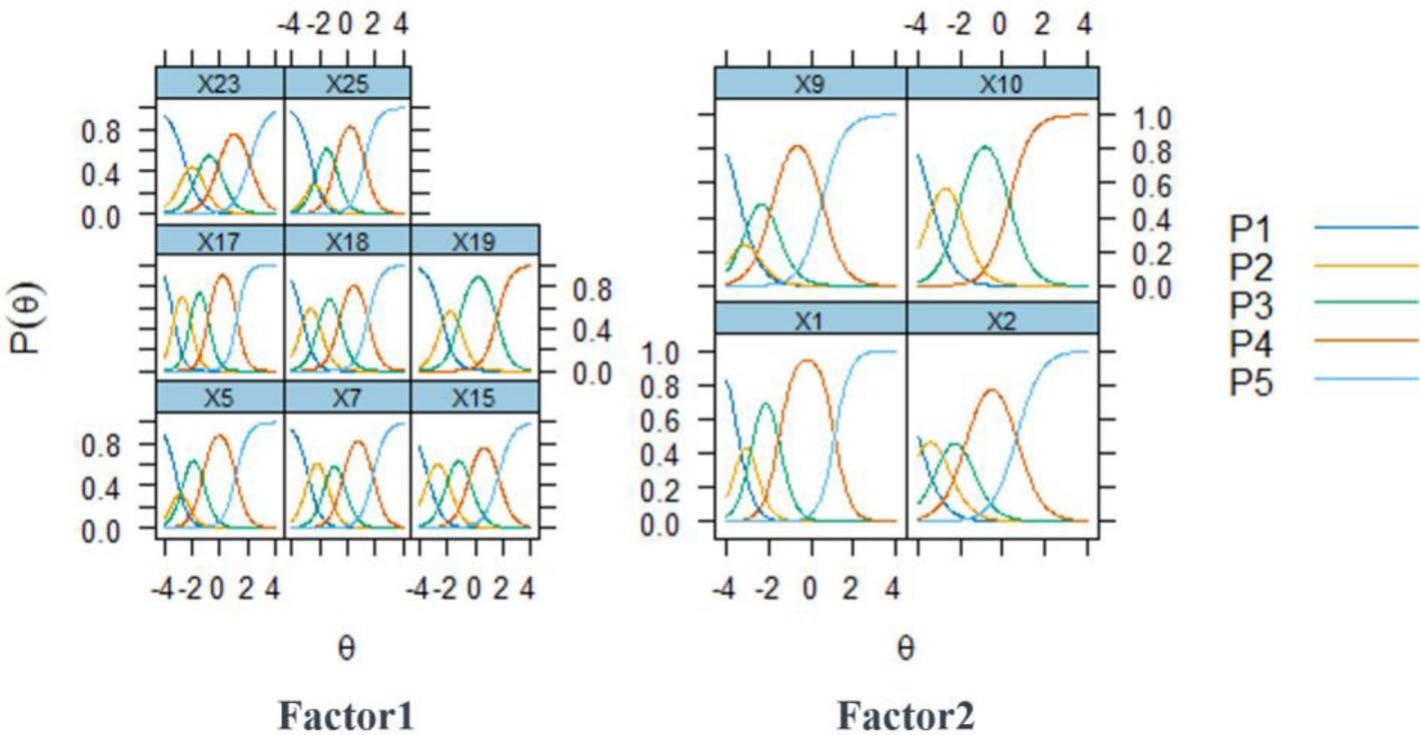
Categorical response curves for each item.

All the items exhibited location parameters that consistently increased with the option levels as can be seen in Fig. 2, with no reversed thresholds noted. The location parameters of the items varied between − 4.014 and 2.118, with items 19 and 10 lacking a b1 location parameter due to responses recorded on a five-point scale with only four options selected. Furthermore, apart from items 7, 23, and 25, all other items displayed first location parameters with absolute values exceeding 3. This highlighted an issue with the option design of these items, with item 2 being the most problematic.

Table 8 revealed the information provided by each item, ranging from 4.34 to 10.38. Factor 1 demonstrated an overall information value of 55.75, whereas Factor 2 exhibited a notably lower value of 22.67, which was less than half of Factor 1's information. Within Factor 1, items 15, 19, and 23 presented relatively lower information values. Notably, items 2, 9, and 10 within Factor 2 had information values even lower than the smallest value observed in Factor 1 (item 23). This further underscored the requirement for significant refinement within Factor 2 as compared to Factor 1.

### Analysis of demographics characteristics

Based on IRT probability analysis, the resilience ability level ($\theta$) in both Factor were calculated and total ability was calculated as 0.67* $\theta ({\text{Factor}} \, 1)$ + 0.33* $\theta ({\text{Factor}} \, 2)$ based on the item quantity.

From Table 9, it was evident that various variables significantly influenced resilience. Greater age, increased flight duration, and relationship stability correlated positively with higher resilience among pilots. Notably, the first officer exhibited significantly lower resilience levels compared to captains and instructors. Surprisingly, instructors also displayed marginally lower resilience levels than captains, which seemingly contradicts conventional wisdom.

**Table 9:** Demographics information.

Group	Sample size	Median (P25, P75)	Kruskal–Wallis–H	p
Age				
21–30	103	− 0.203 (− 0.6, 0.1)	11.475	0.003**
31–40	85	0.110 (− 0.4, 0.6)		
40 +	43	0.110 (− 0.4, 0.9)		
Post				
First officer	119	− 0.203 (− 0.6, 0.3)	10.895	0.004**
Captain	45	0.110 (− 0.3, 0.7)		
Instructor	67	− 0.014 (− 0.4, 0.7)		
Flight experience				
0–3000	88	− 0.199 (− 0.7, 0.1)	11.834	0.003**
3001–7000	56	0.024 (− 0.5, 0.5)		
7000 +	87	0.105 (− 0.3, 0.8)		
Marital status				
Single	28	− 0.325 (− 0.6, 0.1)	15.271	0.002**
In a relationship	37	− 0.210 (− 0.8, 0.1)		
Married without children	43	− 0.203 (− 0.5, 0.5)		
Married with children	123	0.110 (− 0.4, 0.6)		

*p < 0.05, **p < 0.01.

Upon a comprehensive analysis of the data, it was observed that the age distribution among instructors was nearly evenly divided, with approximately half falling within the 30–40 age bracket (n = 27) and the other half in the 40 + age bracket (n = 39). In contrast, the age distribution among captains predominantly centered around the 30–40 age range (n = 34). Initially, a statistical examination of resilience among instructors in these two age brackets was conducted, revealing a slightly higher resilience level among instructors aged 40 + compared to those aged 30–40, aligning with the conclusions drawn based on age. Subsequently, a detailed analysis was performed on the data of captains and instructors aged 30–40, indicating no significant differences in scores between the two groups (p = 0.738). This suggests that, in comparison to the impact of occupational roles on resilience, the influence of age was relatively more substantial.

## Discussions

Resilience stands as a fundamental element crucial for ensuring aviation safety, particularly in high-stress environments like civil aviation, where its absence can significantly impact human-related aircraft accidents. The imperative evaluation of resilience in civil aviation pilots not only aids in identifying individuals requiring increased resilience but also facilitates timely interventions. This study, rooted in measurement data collected from Chinese civil aviation pilots, extensively scrutinized the resilience structure and refined the measurement tool specifically for this cohort.

To our knowledge, this investigation marks the first comprehensive attempt to uncover the underlying factor structure of the 25-item CD-RISC measure within the context of Chinese civil aviation pilots. Furthermore, it pioneers the utilization of both Multivariate Generalizability Theory (MGT) and Item Response Theory (IRT) in the analysis of CD-RISC-25, which significantly enriches the understanding of resilience in this specific domain.

1. Cultural and Occupational Influences on Resilience Measurement tool: Key findings unveiled particular nuances within the CD-RISC-25 items among Chinese civil aviation pilots. Items 3 and 20 displayed incongruities within this population, necessitating their exclusion from the measurement tool. The exclusion of item 3 (“Sometimes fate or God can help”) aligned with many CD-RISC applications in China as well as among American female pilots^16,25^. This may be explained by belief systems in Chinese culture and the occupational characteristics of pilots. Item 20 (“Have to act on a hunch”) also required removal, reflective of pilots’ cognitive demands^54–56^. For pilots, it is not advisable to act based on intuition when faced with unforeseen situations. They are usually advised to take deep breaths before formulating the appropriate strategies. Besides, the analysis revealed significant score differences on the remaining 23 items between groups with varying levels of resilience among Chinese civil aviation pilots. This observation emphasizes the tool's discriminative capabilities in measuring various dimensions of resilience in this specific cohort.
2. Enhanced Understanding of Resilience Structure for Chinese civil aviation pilots: The factor structure analysis unveiled a distinct 2-factor model of resilience among Chinese civil aviation pilots, characterized by "Decisiveness" (Factor 1) and "Adaptability" (Factor 2). Factor 1 focuses on a pilot's self-confidence, decision-making ability, and capability to face challenges, indicating the pilot's high confidence in their abilities and problem-solving skills, and a more resolute and decisive approach when dealing with challenges. Factor 2 centers on adaptability, relational stability, and a positive attitude towards coping with uncertainty and change, demonstrating the pilot's emphasis on coping with change, maintaining close relationships, and adopting an optimistic and persevering attitude when faced with uncertainty. Consistent with most prior research on the factor structure of the CD-RISC, the current study found no support for the original five-factor model^14,16,24^. Comparisons between the present three-factor model and previous findings imply that differences in background factors, such as cultural influences, may have contributed to observed variability in factor structure, since cultural contexts shape resilience awareness and its meaning and structure diverge across cultures. Moreover, our sample did not validate the previously reported Chinese three-factor model. Unlike our focus, Yu et al.'s study sampled individuals in varied occupations^17^, indicating that modifications may be necessary for specific populations. However, compared to the three-factor structure found in the Chinese population^17^, the resilience structure exhibited by female U.S. pilots^26^ demonstrates a better model fit. This might suggest that despite the variations across different cultures, the profession of pilots exhibits a distinct resilience structure different from the general population, warranting individual attention^54,55^.
3. Reliability and Practical Implications of the refined 2-factor measurement tool: Multivariate Generalizability Theory (MGT) surpasses the limitations of Classical Test Theory by providing more robust estimations of reliability while Item Response Theory (IRT) delves deeper into the specifics of each item. The current analysis of the 2-factor test demonstrated strong generalizability and dependability coefficients, signifying its applicability for both norm-referenced and criterion-referenced evaluations. Notably, even with only 5 items retained for Factor 1, strong generalizability and dependability coefficients persist, suggesting the potential for abbreviated questionnaire versions. Furthermore, it was determined that the recommended removal of items for Factor 1 should be 15, 19, and 23. Moreover, correlating the outcomes of the IRT analysis with interviews conducted with pilots, it was observed that, in relation to Factor 2, there exists a higher demand for adjustments in item options compared to Factor 1. This disparity might be attributed to Factor 1's stronger association with flight scenarios, such as emphasizing leadership and emotional stability within flight crew resource management. These elements enable pilots to make more accurate choices^54^. Conversely, Factor 2 exhibits weaker connections to flying, leading to less consistent contextual associations for pilots when answering, highlighting the need for a more flight-oriented adjustment of items within Factor 2 in future revisions.
4. Demographic Correlations: this study uncovered a significant positive correlation between age, flight hours, and relationship stability with resilience. This observation aligned with research on resilience among female aviators in the United States^25^, indicating that the accumulation of professional experience contributes to an enhancement in resilience among pilots. In this study, it was observed that with increasing age, pilots tend to exhibit enhanced psychological resilience. Regarding flight hours, the pilots with longer flight hours in this study generally had a higher age, thereby failing to produce an interaction effect. In terms of occupational positions, an interesting observation was that the resilience of instructors was slightly lower than that of captains. This difference was especially notable among instructors aged 30–40, whose resilience was relatively lower. This phenomenon could be attributed to the fact that, compared to captains, instructors face more communication and coordination challenges^57^. Positioned in the developmental stage of their careers, they might perceive greater responsibilities and pressures, leading to a somewhat lower resilience compared to captains. Furthermore, in the context of marital status, the performance of civilian pilots differed from that of military pilots. Married pilots demonstrated lower resilience, potentially due to the increased family pressure when married resulting from the greater uncertainty associated with military aviation^26^. In contrast, the relative safety of civilian flight tasks enhanced the stability of life brought about by marriage, contributing to the elevated resilience observed in married civilian pilots.

In conclusion, this study contributes a refined and culturally attuned resilience measurement tool tailored for Chinese civil aviation pilots. It not only advances our comprehension of resilience in this specific context but also provides a robust framework for ongoing assessment and interventions, with the ultimate aim of fortifying pilot psychological health and aviation safety.

## Limitations

While this study provided valuable insights into the resilience of Chinese civil aviation pilots, certain limitations exist that warrant consideration and offer avenues for future research within the context of grief and trauma:

1. Population Limitations: The present study primarily focuses on male pilots from different subsidiaries of a Chinese airline. Although these subsidiaries covered various geographical regions within China, it is crucial to acknowledge that different airline companies may employ distinct strategies in cultivating pilot resilience. Therefore, future research should aim to collect data from diverse airline companies to facilitate a comparative analysis of psychological resilience among pilots. Additionally, conducting specific research targeting Chinese female pilots would unveil further and deeper insights into the characteristics of pilot psychological resilience.
2. Limited Exploration of Psychological Correlates: The study primarily focused on demographics in relation to resilience among Chinese civil aviation pilots. Future research should delve deeper into the relationship between resilience and psychological qualities such as psychological fatigue, job burnout, life satisfaction, mental health, and other potential psychological variables. This exploration is crucial for a more comprehensive understanding of how resilience interacts with these psychological dimensions, particularly in the context of coping with potential trauma in the aviation sector.
3. Longitudinal Studies and Intervention Evaluation: While this study focused on the measurement of resilience, future research could benefit from longitudinal studies to assess changes in resilience levels over time, particularly after exposure to distressing events. Additionally, evaluating the efficacy of interventions designed to enhance resilience in the aftermath of grief and trauma would be valuable for further advancing practical approaches in the aviation sector.

## Conclusions

In summary, this research has made significant strides in comprehensively assessing the resilience of Chinese civil aviation pilots, shedding light on crucial elements necessary to consider within the realm of trauma following potentially distressing events. The study's findings contribute to the understanding and assessment of resilience in the following ways:

1. Identification of Culturally and Professionally Incongruent Items: The study reveals discrepancies within the CD-RISC-25 scale for Chinese civil aviation pilots, emphasizing the need for cultural and professional adaptations when assessing resilience. These insights underscore the importance of contextual adjustments in resilience measurement, particularly in the context of high-stress occupations like civil aviation.
2. Development of an Adapted 2-Factor Resilience Model: The establishment of a distinctive 2-factor resilience model, featuring 'Decisiveness' and 'Adaptability', provides a novel framework tailored to the psychological profile of Chinese civil aviation pilots. This model not only offers insights into resilience within this occupational group but also sets a foundation for further research and specialized assessments in the context of trauma following aviation-related events.
3. Utilization of Advanced Measurement Analysis Theories: The study's utilization of Generalizability Theory and Item Response Theory presents a novel approach to scrutinizing and enhancing the CD-RISC-25's applicability in the specific context of Chinese civil aviation pilots. Through the meticulous examination of reliability and item-specific measurement issues, this research opens avenues for improved assessment tools and tailored interventions.
4. Understanding Resilience Variations and Influential Factors: The investigation into variations in resilience based on demographics among Chinese civil aviation pilots offers a crucial understanding of influential elements. These insights serve as a fundamental basis for developing targeted resilience interventions, particularly crucial in the context of potential trauma and grief due to high-stress work environments.

Ultimately, this study emphasizes the necessity of considering cultural nuances and contextual factors when measuring and understanding resilience, particularly in high-stress professions like aviation. The insights and refined measurement tool presented herein pave the way for improved assessment and intervention strategies. These advancements hold the potential to significantly enhance the psychological well-being and safety of aviation professionals, particularly in the context of managing trauma.

## Data Availability

Data available on request from the corresponding author (wanglijing@buaa.edu.cn).
